# Digital technologies: tensions in privacy and data

**DOI:** 10.1007/s11747-022-00845-y

**Published:** 2022-03-05

**Authors:** Sara Quach, Park Thaichon, Kelly D. Martin, Scott Weaven, Robert W. Palmatier

**Affiliations:** 1 Department of Marketing, Griffith Business School, Griffith University, Gold Coast campus, Southport, Queensland 4222 Australia; 2 College of Business, Colorado State University, Fort Collins, CO 80523-1201 USA; 3 Foster School of Business, University of Washington, Box: 353226, Seattle, WA 98195-3226 USA

**Keywords:** Digital technology, Data monetization, Data sharing, Privacy, Social media, Big data, Artificial intelligence, Internet of things, Structuration theory, Privacy regulation

## Abstract

Driven by data proliferation, digital technologies have transformed the marketing landscape. In parallel, significant privacy concerns have shaken consumer–firm relationships, prompting changes in both regulatory interventions and people’s own privacy-protective behaviors. With a comprehensive analysis of digital technologies and data strategy informed by structuration theory and privacy literature, the authors consider privacy tensions as the product of firm–consumer interactions, facilitated by digital technologies. This perspective in turn implies distinct consumer, regulatory, and firm responses related to data protection. By consolidating various perspectives, the authors propose three tenets and seven propositions, supported by interview insights from senior managers and consumer informants, that create a foundation for understanding the digital technology implications for firm performance in contexts marked by growing privacy worries and legal ramifications. On the basis of this conceptual framework, they also propose a data strategy typology across two main strategic functions of digital technologies: data monetization and data sharing. The result is four distinct types of firms, which engage in disparate behaviors in the broader ecosystem pertaining to privacy issues. This article also provides directions for research, according to a synthesis of findings from both academic and practical perspectives.

**Electronic supplementary material:**

The online version of this article (10.1007/s11747-022-00845-y) contains supplementary material, which is available to authorized users.

Modern marketing practice requires the use of digital technologies, and the customer data they generate, to create value (Quach et al., 2020). Yet such reliance prompts increasing privacy concerns about firms’ data behaviors and actions among both consumers and regulators. Consumers thus take action to protect their data; for example, people who switch service providers frequently cite privacy worries as a key reason (Cisco, 2020). However, many consumer respondents to a recent Australian survey (58%) admitted they do not understand what firms do with the data they collect, and 49% feel unable to protect their data due to a lack of knowledge or time, as well as the complexity of the processes involved (OAIC, 2020). Stronger regulations at global, national, and state levels (e.g., Australian Privacy Act, General Data Protection Regulation [GDPR], California Privacy Right Act [CPRA]) may help consumers, but they are costly for firms to comply with (e.g., up to US$55 billion for CPRA, according to estimates by the California state attorney general’s office) and also establish strict penalties for noncompliance (e.g., 10–20 million euros or 2%–4% of global firm revenues for specific GDPR infringements). Thus, privacy concerns create tensions among consumers, firms, and regulators, and effective privacy protection likely requires cooperation among these interconnected groups.

Extensive research details consumers’ privacy concerns (for a comprehensive review, see Okazaki et al., 2020) and regulatory interventions of varying effectiveness (Jia et al., 2021), as well as the consequences for firms’ performance (e.g., Martin et al., 2017). However, we still lack a systematic, integrative, research-based view of privacy tensions across all three involved entities, specifically in relation to digital technologies and the unique customer data they generate (Pomfret et al., 2020). That is, existing research effectively outlines privacy tensions from consumers’ and firms’ perspectives (Bornschein et al., 2020) but without addressing the complex, interrelated positions of firms, consumers, and regulators simultaneously (Martin &; Palmatier, 2020). Research into internal privacy mechanisms such as privacy paradoxes (Kaaniche et al., 2020) or dyadic views of privacy between consumers and firms (Rasoulian et al., 2017) or between firms and regulators (Johnson et al., 2020) cannot establish a triadic view of the privacy tensions created by digital technologies that link all these groups.

Therefore, to develop new marketing insights into digital technologies and privacy, we explicitly consider this firm–consumer–regulatory intersection and work to disentangle the data strategies and embedded technologies that firms use to create mutual value for themselves and their customers. With a comprehensive review of digital technologies, we examine four categories: (1) data capturing; (2) data aggregation, processing, and storage; (3) data modeling and programming; and (4) data visualization and interaction design. Each category can enable data monetization and sharing in distinct ways and with unique implications for consumers’ (information, communication, and individual) privacy outcomes. Accordingly, we investigate the consumer implications of firms’ digital technology use, with a particular focus on their privacy responses. As consumers gain knowledge about digital technologies, they may be more likely to adopt a proactive strategy and take preemptive, protective measures when interacting with firms. Finally, we examine how various regulatory interventions enter into these consumer–firm interactions, by exploring both proactive and reactive regulatory enforcement mechanisms. In pursuing these three research objectives, we establish an integrated framework with relevant implications for consumers, firms, and regulators.

We augment the analyses with case studies (i.e., Apple, Facebook, and BMW) and interview data, gathered from senior managers and consumer informants, which enhance the external validity of the integrated digital strategy framework. In particular, we use informants’ insights to understand people’s growing privacy concerns and the legal ramifications linked to digital technology strategies. Because our findings extend knowledge by blending the perspectives of firms, consumers, and regulators, they also provide meaningful research directions and actionable insights for academics and practitioners. Accordingly, we offer suggestions for research, reflecting the synthesis of the academic and practical perspectives that inform our findings.

This research contributes to marketing theory by applying a structuration theoretical approach to a marketing data privacy context. Structuration theory (Giddens, 1984) overcomes some limitations of prior systems theories that overemphasize the role of either structure or action in social processes and interactions; its theoretical insights instead reflect their interplay. Therefore, it can help us explain how data privacy regulatory frameworks impose structure on consumer–firm–policymaker interactions, then predict reactive and proactive responses by each key actor. The presence (absence) of a regulatory framework provides rules and norms that can mitigate (exacerbate) privacy tensions. In addition to relying on effective regulations for data protection, consumers exhibit other privacy protection behaviors and demands, which then intensify the pressure on firms to respond to privacy tensions.

The findings of this study also help inform marketing practice by delineating firm responses that can offset consumer privacy risks. For example, in some contexts, firm responses to consumer privacy risks are stipulated by a well-defined regulatory mandate, though even in this case, they may be subject to multiple, conflicting regulations (Lavelle, 2019). In unregulated settings, firms must self-police to meet privacy expectations, despite a lack of insights into how to mitigate the threats and risks of privacy failures (e.g., data breaches, data misuse scandals). Another option would be to exceed regulatory stipulations and use privacy as a source of competitive advantage (Palmatier & Martin, 2019), in which case firms need specialized knowledge of how to infuse privacy proactively into all their structures and processes. Noting these options, we provide practical advice for how firms can adopt a reactive stance and respond to privacy mandates on an as-needed basis or else become more proactive by exhibiting privacy-by-design, zero-party data collection, or ecosystem innovation, among other approaches.

In the next section, we begin with a description of the consumer privacy tensions that emerge from firms’ digital technology uses in four areas: data capture; data aggregation, processing, and storage; data modeling and programming; and data visualization and interaction design. We then review these conceptualizations from a structuration theory perspective, from which we derive some suggested proactive and reactive responses for regulators, firms, and consumers. Using this discussion as a foundation for our integrative framework, we offer three thematic tenets and seven research propositions, which can inform a comprehensive firm data strategy typology, as well as an extensive research agenda.

## Firms’ digital technology use and consumer privacy tensions

Digital technologies allow firms to access vast amounts of data, which they might leverage to increase their profitability (i.e., data monetization) or improve the performance of their broader business networks (i.e., data sharing). Specifically, *data monetization* means the firm exploits data for their direct or indirect economic benefits. These practices might include applying data analytics–based insights to develop new products and services for the customers whom the data represent (i.e., data wrapping). For example, Coca-Cola collects data to improve customer service and performance, such as development of a Cherry Sprite flavor, based on data collected from self-service vending machines and social monitoring empowered by AI-driven image recognition technology. Data monetization also involves harnessing insights to create value-added features for other clients (i.e., extended data wrapping). Facebook, for example, makes money by providing data analytics features to advertisers based on user data on its social network platform. Finally, a direct approach to data monetization is for firms simply to sell their data to other firms (Najjar & Kettinger, 2013). Comscore is a digital analytics organization that provides marketing data and information to advertisers, media and marketing agencies, publishers, and other firms, selling these data to more than 3200 clients in 75 countries.

*Data sharing* instead refers to resource exchanges in which firms provide data they have gathered to various network partners (e.g., suppliers, distributors, horizontal partners with complementary offerings), to facilitate collaboration in the broader ecosystem (Sydow & Windeler, 1998). For instance, Coca-Cola shares information with third parties such as hosting firms, IT service providers, or consultants that support its service provision. Coca-Cola’s EU website (https://www.coca-cola.eu/privacy-notice/) lists 18 third parties with which it shares data. PayPal, by contrast, lists 600 such parties. Other tech firms such as Apple work with complex networks of suppliers and application developers that constantly exchange information to develop better products and services. In 2018, a New York Times investigation revealed that Facebook shared data with more than 150 companies. Such data-based collaborations improve the performance of its entire digital ecosystem. Thus, data monetization increases firm profitability more directly, whereas data sharing improves profitability via network performance.

Levels of data sharing and data monetization vary across firms (see Web Appendix 1). For example, *data harvesters* are mostly firms in non-technical industries that engage in very limited data sharing and data monetization. Few harvesters engage in data wrapping, which would demand significant investments in digital technologies. Many of them are small firms with low to moderate levels of digital competence, though others are huge firms that recognize their own data insights are more valuable than any data they might purchase from outside sources (e.g., Coca-Cola, adidas, McDonald’s). *Data patrons* (e.g. Apple, Paypal) often possess moderate to high levels of digital technology and invest in sharing data across networks of partners, such as suppliers and distributors, to improve the overall functioning of the ecosystem. Even if they share data extensively, they also impose strict limits on how those data can be used and if (whether) they may be monetized. On the other hand, *data informants’* business models rely on extensive data monetization and include data brokers, app developers, and content creators (e.g., Comscore, Weather Bug, OnAudience). With vast digital technologies, they generally engage in little sharing but monetize data through extended data wrapping (e.g., game development services) or sales of information or analytics (e.g., data brokering services). *Data experts* (e.g. Facebook, Google) engage in high levels of both data sharing and data monetization. Due to their significant digital technology resources, they own a lot of data and also control most of the data flows in the digital ecosystem. They predominantly perform extended data wrapping to attract new customers. That is, data experts offer their customers’ data to other clients, such as advertisers, that use the insights to reach their own target customers.

Data sharing and monetization practices generally involve a diverse portfolio of digital technologies, each of which can create benefits but also trigger privacy tensions, as we describe next and summarize in Table 1.

**Table 1:** Digital technology tensions and consumer privacy risks

**Data Strategy (Firm)**		**Privacy Risks (Consumers)**		
**Data sharing** *allowing firm partners or outside entities to access or use a firm’s data*	**Data monetization** *extent to which the firm uses data for its own economic benefit*	**Information privacy** *consumers’ right to control the access to, use, and dissemination of their data*	**Individual privacy** *right of a person to be left alone without disruption*	**Communication privacy** *protections for communications against interception and eavesdropping*
**Data capturing technologies: Main sources of consumer information**				
***Social media****(gathering demographics, psychological, geographic, and behavioral data) (*de Oliveira Santini et al., 2020*;* Kamboj et al., 2018*)*				
• Social media rely on user-generated content, and consumers voluntarily share substantial personal information and other useful insights through these technology platforms. Data collected from social media might be shared with partners, such as members of the business network, for better market insights and data-based innovation.	• ***Marketing and operational performance:*** tailored content based on customer profiling to develop relationships; targeted advertising to maximize conversions. • ***Potential for data wrapping/extended wrapping:*** data can be used to develop analytics-based features and experiences that inspire customer actions, such as for the benefit of advertisers or app developers. • Data might be sold to third parties, such as advertisers.	• Being unable to control the flow of information. • Third parties’ access to profile information and user-generated content from well-developed application programming interfaces.	• Organizations might be able to reach consumers through location disclosures, such as tagging a venue in their posts on social media.	• Risk of exposing information of close ties; firms might intercept and exploit the exchange between two connected contacts.
***Geospatial technology****(using technologies such as geographic information systems, geofencing, and GPS to collect location data) (*Sun et al., 2015*;* Zubcsek et al., 2017*)*				
• Data and location insights might be shared with partners, such as members of the business network, for better market insights and data-based innovation.	• ***Marketing and operational performance***: location-based marketing; customer profiling and personalization; optimization of distribution network and maximize retail performance. • ***Potential for data wrapping/extended wrapping:*** location analytics such as navigation, directories, and traffic updates. • Data might be sold to third parties such as advertisers.	• Confidentiality of accumulated location data, disclosing both travel history and real-time position of an individual.	• Organizations are able to pinpoint the exact locations of users and reach them. • Signaling surveillance.	
***Biometrics****(collecting physiological and behavioral data that allow for precise recognition capabilities) (*Ioannou et al., 2020*;* McStay*,*^2020^*)*				
• Data might be shared with partners, such as members of the business network, for better market insights and data-based innovation.	• ***Marketing and operational performance:*** customer profiling; data can be used to develop authentication systems (e.g. FaceID) and streamline business processes (e.g. facial recognition based boarding solutions). • ***Potential for data wrapping/extended wrapping:*** biometric data can be used to develop analytical features that optimize user experiences such as medical alerts. • Biometrics data might be sold to third parties that use them for various purposes, such as product development.	• Lack of control over the use of highly sensitive and immutable information, which can reveal a person’s identity. • Objectification of emotions and manipulation.	• Biometric data are vulnerable to hacking and coveted by cybercriminals, which increases the potential for identity theft, stalking, and disruption to personal lives.	
***Web tracking****(collecting digital footprints with online tracking technologies such as cookies, flash cookies, and web beacons) (*Sabillon et al., 2016*;* Zarouali et al., 2017*)*				
• Data might be shared with partners, such as members of the business network, for better market insights and data-based innovation.	• ***Marketing and operational performance:*** customer profiling, market segmentation, personalization and retargeting. • ***Potential for data wrapping/extended wrapping:*** data can be used to develop analytical insights for advertising services offered to advertisers. • Information may be readily sold, so external firms can exploit deep knowledge of consumer browsing behavior.	• An extensive profile of customers can be built by tracking their visits to multiple websites, which defies anonymity. • Information might be shared with third parties. • These technologies are often hidden and hard to detect or delete.	• Individuals can be followed by using their digital footprints.	
**Data aggregation, processing, and storage technologies: Combining data from multiple sources and developing actionable analytics**				
***Internet of Things****(connected devices that exchange significant amounts of data in machine-to-machine communications) (*Kobusińska et al., 2018*;* Palmatier & Martin, 2019*)*				
• Access to real time data through connected devices.	• ***Marketing and operational performance:*** relationship development with customers; real-time insights for customer profiling and behavior prediction; customer engagement; augmented experiences with cross-device features; increased firm efficiency, responsiveness, and proactivity. • ***Potential for data wrapping/extended wrapping:*** cross-device data analytics-based features can be developed. • Data might be sold to third parties	• Sensitive information may be collected and shared in real-time among different IoT-enabled systems and devices. • Lack of control over data access and exchange, especially in machine-to-machine interactions. • Smart devices are very vulnerable to cyberattacks.	• Firms or third parties might reach customers using IoT-enabled devices and systems without being noticed, such as with CCTV cameras that track people using facial recognition technology.	• IoT-enabled devices and systems can capture and transmit communications between users, such as when integrated microphones capture conversations. • The IoT devices seize data from not just users but also proximal others.
***Big data****(large volumes of high velocity, complex, variable data) (*Kopalle & Lehmann, 2021*;* Park et al., 2018*)*				
• Insights and analytics might be shared with partners, such as members of a business network.	• ***Marketing and operational performance:*** customer profiling; personalization and prediction of customer demand and accurate targeting; optimization of business operations and supply chain management. • ***Potential for data wrapping/extended wrapping:*** accumulation of data can be used to develop data analytics-based features for a product/service. • Insights and analytics might be sold to third parties.	• Identifiable information and highly sensitive personal attributes such as sexual orientation, age, and political views may be collected. • Algorithmic profiling and aggregation leads to a comprehensive picture of an individual. • Unauthorized access and lack of control over the accumulated information.	• Risk of stolen identity, violation of personal spaces, and loss of intellectual property. • Being subject to sophisticated manipulation using predictive analytics. • Potential discrimination from customer profiling, which increases individual vulnerability.	• Private communications might be captured from different data points using data mining tools.
***Cloud****(storage and analytics) (*Alsmadi & Prybutok, 2018*;* Yun et al., 2019*)*				
• Access to data, applications, and services by multiple users in real time; data storage at reduced technology costs.	• ***Marketing and operational performance:*** optimization of business performance and supply chain management through on-demand services and handling big data; data storage at reduced technology costs. • ***Potential for data wrapping/extended wrapping:*** cloud computing provides massive storage and computing capabilities to customize user experiences with data. • Data and analytics might be sold to third parties.	• High risk of unauthorized access due to virtualization and remote processing and storage, especially during the transmission of data across different platforms. • Data leakage often results in significant data losses. • Risk of information exposure to external groups such as fourth parties. • Cloud service providers are often private firms, raising questions about data access, control, availability, and backup.	• Firms or third parties might be able to track customers using real-time data stored in cloud services.	• Private communications in cloud storage might be intercepted.
**Data modeling and programming technologies: Automation of tasks and services**				
***Artificial intelligence/machine learning****(intelligence exhibited by machines or software capable of performing human tasks) (*Davenport et al., 2020*;* Kwok & Koh, 2020*)*				
• Enabling automated sharing of real-time data.	• ***Marketing and operational performance:*** personalized recommendations and content; more effective, efficient and transparent programmatic advertising; cost reduction in media production using deepfakes; predictive models of customer behavior; retargeting strategies; improved operational efficiency due to automation. • ***Potential for data wrapping/extended wrapping:*** AI-powered systems can produce data analytics-based features that can act and adapt automatically. • Insights and analytics might be sold to third parties.	• It has become very easy and inexpensive to identify, profile, and manipulate consumers without their consent. • Enormous amounts of data are required to train AI, often unnoticed by customers. • AI has the ability to predict sensitive data based on seemingly harmless pieces of information.	• Information may be used to produced fake content (e.g., deep fakes) to manipulate customers or reach them instantly.	• Advanced AI agents can interact with users and make sense of the conversations between them.
***Service robots****(embodied AI blending engineering and computer science) (*Mende et al., 2019*;* Xiao & Kumar, 2019*)*				
• Enabling automated sharing of real-time data, some of which might be from physical interactions.	• ***Marketing and operational performance:*** customer assistance and service; improving customer experience; increasing organizational efficiency and effectiveness due to the automation of tasks and services. • ***Potential for data wrapping/extended wrapping:*** AI-powered systems embedded in robots can produce data analytics-based features that can act and adapt automatically to real-time, physical environments. • Data and analytics might be sold to third parties.	• Robots’ autonomy means humans have less control over their data. • Third parties’ management and usage of personal information may change after multiple iterations of data.	• Potential intrusion into physical and emotional space due to physical and personal contact with robots.	•Robots equipped with computer vision and machine learning see and sense the environment; can analyze human characteristics e.g. age, gender, emotions; and can make sense of humans’ conversations.
**Data visualization and interaction design technologies: Interaction with multidimensional data**				
***Mixed, augmented, and virtual realities****(convergence of physical and digital environments through computer-generated simulations involving synthetic worlds)(*Hilken et al., 2017*;* Nijholt*,*^2021^*)*				
• Access to data through connected realities; visualizations and data storytelling can be shared quickly and seamlessly across groups of users.	• ***Marketing and operational performance:*** omnipresence and seamless experience; development of intimate and meaningful relationships with customers; innovative platforms for social media marketing; increased firm efficiency, responsiveness, and proactivity through immersive analytics. • ***Potential for data wrapping/extended wrapping:*** data analytics-based features can be depicted for easy access and immersive experiences. • Insights and analytics might be sold to third parties.	• Sensitive, real-time information and private communication can be captured by input devices. • Both output and input devices can communicate wirelessly, resulting in a lack of control over the collected information.	• Physical space might be captured, such as by spatial mapping of information when people engage in mixed or augmented reality, including bystanders. For example, social AR in public spaces likely captures passers-bys’ facial and behavioral data, without them noticing. • Output data might be exposed to other parties and manipulated to deceive users, such as in clickjacking practices.	• Personal communications can be captured by devices such as cameras and microphones.

### Privacy tensions

Digital technologies offer data monetization and sharing benefits to firms but have concomitant costs for consumers, especially with respect to privacy. Westin (1967) defines privacy as a person’s right “to decide what information about himself should be communicated to others and under what condition” (p. 10), whereas Altman (1975) regards it as “the selective control of access to the self” through social interactions and personal space (p. 24). Adopting these definitional premises of autonomy, access, and control, we conceive of three types of consumer privacy: information, communication, and individual (see also Hung & Wong, 2009). The simultaneous consideration of all three types offers an expansion of extant marketing studies of privacy that tend to focus solely on information privacy (Bornschein et al., 2020). In detail, *information privacy* refers to a consumer’s right to control the access to, use, and dissemination of her or his personal data (Westin, 1967). Thus people may decide for themselves when, how, and to what extent their information will be known by others. *Communication privacy* protects personal messages or interactions from eavesdropping, scanning, or interception. People generally prefer to keep their interpersonal communications confidential and safe from third-party surveillance, which would not be possible if conversations with friends were recorded by social media and messaging apps or their in-person discussions were captured by smart devices equipped with integrated microphones. Finally, *individual privacy* is being left alone without disruption (Westin, 1967). Threats to individual privacy involve personal space intrusions, emotional manipulation, and physical interference, including spam emails and retargeting practices. Such violations are on the rise, due to the presence of IoT and smart home devices installed in consumers’ personal, physical spaces. In turn, firms’ data strategies, enabled by digital technologies, have implications for each type of consumer privacy.

Extensive research details consumers’ privacy concerns (e.g., Okazaki et al., 2020), as well as some of the consequences for firm performance or regulatory interventions. However, we still lack a systematic understanding of how privacy issues arise from firms’ data strategies and their uses of various digital technologies to support such strategies. To articulate the critical tensions between firms’ technology uses for data sharing and data monetization purposes, and consumers’ privacy risks, we combine the three forms of privacy with the data sharing and data monetization strategies related to four digital technology classifications: (1) data capturing; (2) data aggregation, processing, and storage; (3) data modeling and programming; and (4) data visualization and interaction design (Table 1). It would be impossible to discuss all technologies; rather, we attend specifically to six broad groups of emerging technologies: SMAC (social media, mobile, analytics, cloud), digital surveillance, robotics, AI, IoT, and mixed (virtual, augmented) realities (VR, AR). Each of these is characterized by consumer-marketer interactions and is central to firms’ data monetization and sharing strategies (Poels, 2019). Digital technologies such as blockchain, digital fabrication (e.g., 3D printing), 5G, and quantum computing are beyond the scope of this study, because they mainly support operations and digital infrastructure functions.

### Data capture privacy tensions

Data capture technologies, including various sources and methods of data extraction, fuel data sharing and data monetization practices. In this respect, instead of technologies that collect transactional data such as point-of-sale systems, we focus on *social media*, *geospatial*, *biometrics*, and *web tracking* technologies. To facilitate data sharing, the data gathered via these technologies can be shared readily with business partners and networks, such as between manufacturers and suppliers or across subsidiaries (e.g., WhatsApp shares phone numbers, device specifications, and usage data with other Facebook [recently rebranded to Meta] companies). The data collected from social media, geospatial, biometrics, and web tracking technologies can also be monetized in various ways. With user-generated social media content, location insights from geospatial technologies, biometric data, and web tracking technologies such as cookies, firms can improve marketing and business performance by developing market segmentation and (re)targeting strategies, by crafting personalized content, products, and experiences, and by building and strengthening customer relationships (de Oliveira Santini et al., 2020). They also can conduct data wrapping, for example, through customization and optimization practices such as facial recognition and medical alerts (e.g., Apple watch). Firms also can apply extended data wrapping or sell data to other entities. Facebook, as noted, sells in-depth insights and analytics based on its users’ personal data (Appel et al., 2020), and Twitter sells third-party subscriptions to its API that allow other firms to explore users’ behaviors.

These practices threaten *information privacy* because consumers lose control over who has access to their personal information and communicative exchanges (e.g., tweet, review on a public Facebook page). Geospatial data enable firms to identify customers’ positions; by monitoring consumers’ digital footprints, companies also can follow them across different platforms, raising concerns about *individual privacy*. Soft biometric data, about moods or emotions, raise security and ethical concerns, because they reflect personal feelings that can be manipulated for commercial purposes, which would represent individual privacy violations*.* Each user’s information might also include details about other users, due to the networked nature of social media. If a user tags a friend on a public Facebook post, their conversations get exposed, which violates both friends’ *communication privacy* if firms review and exploit these exchanges.

### Data aggregation, processing, and storing privacy tensions

Firms often combine data sets from multiple novel sources, which allows them to effectively share and monetize such data. Key technologies in data aggregation, processing, and storing technologies are *IoT, big data,* and *cloud computing*, with capacities to process and manage massive amounts of information (Kobusińska et al., 2018). The convergence of IoT, big data, and cloud computing is central to data sharing as it enables firms to share applications and analytics with multiple parties in real-time and at reduced technology costs. Data can be shared via IOT-enabled devices in machine-to-machine communications. Insights and analytics based on big data can be exchanged with partners, whereas cloud technologies offer a cost-effective information storage cyber-infrastructure that is broadly available across time and space and accessible by multiple users simultaneously (Alsmadi & Prybutok, 2018). Data aggregation, processing, and storing technologies empower data monetization practices by establishing novel insights about customers from IoT-enabled devices and big data, facilitated by cloud technologies, which can inform consumer profiling, behavior prediction, and targeting efforts. In turn, these efforts can optimize marketing and business performance, supply chain management, and (extended) data wrapping (i.e., development of analytical functions). Accordingly, these technologies have been widely adopted by many businesses, such as Netflix (Izrailevsky et al., 2016) and Woolworths (Crozier, 2019), to improve their performance and profitability.

Both data sharing and monetization practices in this domain can result in significant privacy tensions. Data collected from IoT devices such as CCTV cameras that track people using facial recognition technology and wearable devices that gather real-time information about users’ medical conditions or physical activity are very sensitive and highly personal. A comprehensive personal picture created through data aggregation and algorithmic profiling using big data analytics increases *information privacy* concerns, because it can reveal identifiable attributes such as sexual orientation, religious and political views, and personality (Kshetri, 2014). Moreover, when their behavior can be predicted more accurately, consumers become more susceptible to marketing efforts. For example, gambling companies might pinpoint addicts and entice them with free bets (Cox, 2017). Less purposefully, cloud services rely on virtual storage, but such remote processing can compromize system security (Alsmadi & Prybutok, 2018), especially at the transition moment, when firms shift internal applications and data to the cloud, which risks information exposure to fourth parties, including unethical actors that seek to steal consumers’ personal data (Yun et al., 2019). The sheer volume of information, historical and real-time, that links connected consumers, especially those proximal to one another through IoT devices, heightens security risks involving stolen identities, personal violations, and intellectual property losses (Kshetri, 2014). These practices together threaten *communication privacy* and *individual privacy* because they are intrusive, invisible, and extraordinarily difficult to control.

### Data modeling and programming privacy tensions

Automation enabled by data modeling and programming technologies plays a key role in data sharing and data monetization. Considering our focus on privacy tensions, we discuss *AI/machine learning* and *service robots* as relevant amalgamations of engineering and computer science that produce intelligent automation, capable of learning and adaptation (Xiao & Kumar, 2019). These technologies facilitate data sharing as AI generally enables automated sharing of real-time data, and embodied AIs such as robots can exchange information in physical interactions. Moreover, AI-based systems enable data monetization by improving marketing and operational performance (e.g., personalized recommendations, smart content, programmatic media buys, chatbots, and predictive modeling) (Davenport et al., 2020). Modern robots, such as humanoid, programmable Pepper (Musa, 2020), can understand verbal instructions, interpret human emotions, and exhibit social intelligence to improve customer experiences and optimize performance. AI and service robots also enable data wrapping/extended wrapping by automating tasks and services; in addition, their data analytics–based features can adapt automatically to the real-time, physical environment.

However, optimizing machine learning requires enormous amounts of data, collected from consumer interactions, often without their knowledge. In general, AI might extract sensitive information such as people’s political opinions, sexual orientation, and medical conditions from less sensitive information (Davenport et al., 2020), then manipulate users through predictive analytics or create deception such as deep fakes (Kietzmann et al., 2020), which threaten *information privacy*. Robots equipped with computer vision and machine learning both see and sense the environment, implying greater penetration into consumers’ private, physical, and emotional spaces and threats to *individual* and *communication privacy*.

### Data visualization and interaction design privacy tensions

Finally, data sharing and monetization activities rely on data visualization and interaction design technologies, as each enables connected realities known as the “metaverse,” predicted to become an important part of digitial future (Kim, 2021). Data can be visualized through display technologies, such as *mixed, augmented (AR),* and *virtual (VR) realities*, which deliver realistic virtual experiences involving synthetic worlds in which users become immersed through interactions and sensory stimulation (Roesner et al., 2014). In terms of data sharing, these technologies allow immersive data presentations and experiences, especially data storytelling, that can be shared virtually, visually, and seamlessly among different groups of users (customers). In addition, these technologies enable firms to monetize data because they enhance customer interactive experiences (Hilken et al., 2017); they allow marketers to build increasingly intimate customer relationships, as in the examples of Sephora’s virtual product try-on or Facebook’s social VR platform Horizon (Appel et al., 2020), thereby improving marketing and operational performance. Both VR and AR technologies offer great potential for data wrapping/extended wrapping by realistically depicting analytics-based features.

Privacy tensions created are similar to those created by the IoT. Notably, alternate realities require sophisticated input from cameras, GPS, and microphones to enable the simultaneous functioning of various applications (Roesner et al., 2014). Blending mixed reality also requires sensitive information, such as personal communications, images captured by cameras, and movements captured by sensors, posing a risk to *information* and *communication privacy*. Some of the latest privacy concerns involve bystanders in social AR in public spaces, because the data of passers-by, such as their faces or behaviors, can be captured by AR devices without their realization (Nijholt, 2021). The processed data then could be transferred to other applications for display or rendering too, such that their personal information is exposed to an unknown system that might access and manipulate the data without users’ consent. “Clickjacking” tricks people into clicking on sensitive features by using transparent or deceptive interfaces, which then allows the illegitimate actor to extract their data (Roesner et al., 2014). Finally, an extensive range of sensitive sensors can capture rich information, as when visual data produce spatial mapping information also validate spatial elements, such as exteriors or physical articles. Such exposures of physical space threaten *individual privacy*.

## A structuration approach to digital technology–privacy tensions

Data monetizing and data sharing, achieved through firms’ use of digital technologies, can exacerbate technology–privacy tensions among consumers, regulators, and firms. Underpinned by structuration theory, we advance a framework for understanding their unique approaches to managing such tensions in Table 2.

**Table 2:** Privacy responses among consumers, regulators, and firms

		Reactive	Proactive
Consumer data protection behavior	*Information*	• **Falsification:** provide fake information in public posts or when asked by online service providers • **Avoidance:** refuse to provide information • **Self-censorship:** delete or edit past posts; contact the company to remove personal details; remove tags or unfriending	• **Restraint:** minimize user-generated content, such as social media posts and comments • **Encrypted communications:** email encryption or anonymous re-mailers; passwords for sensitive documents/data • **Non-digital alternatives:** face-to-face, traditional media
	*Permission*	• **Withdrawal:** remove cookies from browsers and computers; adopt ad blockers; delete apps when asked for information • **Fortification of identification:** change passwords after data breaches • **Communication termination:** opt-out from mailing lists and from other communications	• **Screening:** check server security (i.e., https); check the privacy policy • **Restriction**: turn off location-based access; change cookie settings • **Identity masking:** private browsing; virtual private networks (VPNs); The Onion Router • **Security consolidation:** use privacy-enhancing technologies such as pop-up window blockers, firewalls, and other internet security programs
Data privacy regulation	*Privacy policy*	• **Availability and visibility** of the privacy policy	• **Specification and promotion of consumer rights** in relation to data privacy
	*Managerial practices, enforcement*	• **Penalty** for non-compliance • **Disclosure** of data breaches to customers and regulators	• Obtain consumers’ **consent** for the collection, use, and dissemination of personal information • Provide consumers **access** to their own data and right to opt out, request to remove their data, or stop sharing it with third parties • **Privacy impact assessment and data protection governance**
Firm responses	*Privacy approach*	• **Local approach:** aim to meet specific, local privacy regulations and laws	• **Universal approach:** tackle global privacy framework in a coordinated manner, and in anticipation of the changes in the overall regulatory framework, often targeting the most restrictive legal requirements
	*Privacy process*	• **Privacy as a feature:** privacy is only a value-added component of a product/service • Improve **data security** by investing in cyber security technologies such as two-factor authentication, encryption, and tokens. • Automated and standardized procedures to facilitate the **removal, transfer, or recovery of data**, especially upon customers’ request.	• **Privacy by design:** privacy is embedded in all business processes, products, and services from the beginning to the final stage; security and privacy are default options for consumers • **Data collection:** zero party data • **Data discovery, categorization, and flow mapping:** categorizing types of data to ensure that firms only collect data that they actually need • **Ecosystem innovation:** involving third parties in data governance policy for more accountable business practices

As noted previously, structuration theory highlights the interaction of structure and action (agency) rather than remaining limited, as some previous social theories had been, to the exclusive role of just structure or action (Giddens, 1984). It thus advances a structural duality account, involving the mutual interdependence and recursivity of actions and structures. Structures, which represent both the context and the outcomes of social practices (Luo, 2006), include rules, laws, social norms, roles, and resources (e.g., digital technology), such that they might constrain or enable (group and individual) agents’ behavior (Jones & Karsten, 2008). Structuration theory also predicts the production and reproduction of a social system through interactions by actors bound by the structure. These actors rely on rules and resources to define meaningful action (reactive approach) and also might extend or transform rules and resources (proactive approach) through their actions (Sydow & Windeler, 1998). Firms and consumers inherently belong to social systems that establish structures, such as regulatory frameworks or strongly held social norms about privacy. Privacy tensions also stem from social practices that evoke responses from consumers and firms. Therefore, even as consumers and firms are influenced by regulatory frameworks and privacy norms, their actions inform and shape those regulatory frameworks and norms. This iterative, dynamic interplay establishes the rules that govern subsequent interactions, forming and refining policies and constraints (Park et al., 2018).

For analytical purposes, Giddens (1984) characterizes structure according to three dimensions: signification (meaning), legitimation (norms), and domination (power). Then interactions consist of three corresponding characteristic forms: communication, (exercise of) power, and (application of) sanctions. Separate modalities connect structure and action. In practice, these elements often are interconnected and function simultaneously (Giddens, 1984). Considering the novelty of this structuration theory application to privacy topics, as well as the complexity of our proposed model, which involves interplays of institutions (regulators), groups (firms), and individuals (consumers), we focus here on the duality of structure and social practices in an effort to clarify privacy tensions among firms, consumers, and regulators, rather than test the original analytical dimensions of structuration theory.

When considering digital technologies and privacy tensions, the structure–actor relationship also might be described according to the service-dominant logic (SDL), which indicates that actors do not function in isolation but are part of wider networks (Vargo & Lusch, 2016). A firm ecosystem comprises a web of strategic networks, in which actors are connected and exchange resources to cocreate value, within the constraints of relevant institutions or institutional arrangements (regulatory authorities, frameworks) (Roggeveen et al., 2012). Firms operate within ecosystems and continuously interact with other entities such as supply chain partners. Because data constitute a type of currency in the digital economy, they represent important elements in any firm’s value chain and the broader marketing ecosystem. By integrating structuration theory with the SDL, we can derive a framework of relationships among actors (regulators, consumers, firms) and relevant structures or institutions (Vargo & Lusch, 2016). This blended perspective implies that the actors exist and interact within a system of relationships (Giddens, 1984; Roggeveen et al., 2012). Accordingly, we can explain the regulatory framework associated with privacy (i.e., structure) and predict both reactive and proactive responses by consumers and firms (i.e., actors). As we noted previously, the presence (or absence) of a regulatory framework implies rules or norms that in turn affect privacy tensions. In addition to relying on effective data protection though, consumers engage in further protective behaviors and demand data protection, forcing firms to respond to the privacy tensions.

### Data privacy regulation

According to structuration theory, structures such as regulatory frameworks (i.e., rules) can both constrain and enable consumer and firm actions in the digital landscape, exacerbating or offsetting privacy tensions. Privacy regulatory frameworks or policies seek to provide fairness, trust, and accountability in consumer–firm data exchanges. Similar to other consumer-focused public policies, major privacy frameworks attempt to improve overall societal well-being and protect people’s rights, in balance with countervailing societal goals such as firm profitability and economic prosperity (Davis et al., 2021; Kopalle & Lehmann, 2021). Digital technologies have evolved significantly, smoothing processes that allow firms to monetize and share customer data while simultaneously adding complexity to consumer-side privacy prevention. Therefore, it is critical for regulators to address privacy tensions that arise from digital technology use.

The three broad classes of privacy risks created by firms’ data monetization and sharing strategies are addressed to varying degrees by global data protection laws such as the GDPR, Australian Privacy Act, and CPRA, each of which attempts to limit the collection, use, storage, and transmission of personal information. Although data privacy regulations differ from country to country, the GDPR has become a global standard (Rustad & Koenig, 2019). New privacy laws tend to reflect its foundations (Bennett, 2018), and the global nature of business implies that many international firms must comply with its rules. Most U.S. state-based and global data protection frameworks share three common principles as their foundation (Helberger et al., 2020), which also align with structuration theory themes. First, consumers are both content receivers and data producers, making consent and ownership critical. Second, transparency is paramount to balance power discrepancies between consumers and firms. Third, data move throughout marketing ecosystems and across multiple parties, making access and control of data streams and consumer education about data collection, uses, and potential consequences critical.

Data protection laws also tend to involve two main enforcement methods, related to firms’ privacy policies and managerial practices, which might be categorized as more reactive or more proactive. Reactive conditions imply minimal changes and less impact on existing firm structures and performance; proactive conditions require more expansive changes. In relation to a firm’s privacy policy, for example, a *reactive* requirement might stipulate its availability and visibility on the firm’s website. For example, CPRA requires a privacy policy hyperlink on the firm’s home page that is noticeable and clearly identifiable (e.g., larger font, different design than surrounding text). A *proactive* version might require firms to disclose consumers’ rights and information access, use, and storage rules as important elements of their privacy policy. Through either enforcement mechanism, the regulatory goal is that consumers learn easily about data security, data control, and governance measures enacted by the firm.

In terms of managerial practices, a reactive approach would mandate notice of data breaches. Most data protection laws also set penalties for noncompliance; under GDPR, firms convicted of privacy violations face fines of up to 20 million euros or 4% of their global revenue. A proactive version might require firms to obtain consumer consent for information collection and usage. For example, websites often use a pop-up window that details the different types of cookies used for tracking and parties with which data may be shared. Consumers may review this information, then opt-out or request that the firm delete their information or stop sharing it with third parties. Both GDPR and CPRA enforce these consumer protections. Other regulations address firm profiling practices, facilitated by AI, to prevent harmful consumer alienation or exclusion practices. However, such laws differ in notable ways. For example, under the GDPR, firms must conduct a regular privacy impact assessment, which is not required by CPRA.

## Consumer privacy protection behavior

Structuration theory suggests that as consumers grow more aware of various privacy tensions during interactions with firms, their sense of worry or fear might evoke protective actions (Walker, 2016). The level of fear or worry depends on the nature of the rules and resources available in their relationships with firms. Assessments of relationship structures likely refer to the severity of the privacy risks, their perceived likelihood*,* and felt vulnerability or agency to cope with privacy risks (Lwin et al., 2007). For example, if consumers realize greater privacy risks due to the nature of the data being collected or increased breach likelihood in a firm relationship, they become more likely to engage in privacy protective behaviors, manifested as future responses to the structures and resources available within that relationship.

Some privacy-protecting strategies increase consumers’ control over personal information (e.g., decrease disclosures, minimize their digital footprint) or establish requirements for explicit permission for uses of their personal data (information access and use) (Walker, 2016). Thus, we again can identify reactive and proactive protection strategies. With a proactive strategy, consumers preemptively address privacy threats; with a reactive strategy, they act as explicitly advised by a firm or in response to an immediate threat. Therefore, we propose a two-dimensional categorization of consumer privacy protection behavior that spans reactive/proactive and information control/permission control dimensions and produces four groups (see Table 2): (1) reactive information strategy, (2) proactive information strategy, (3) reactive permission strategy, and (4) proactive permission strategy.

### Reactive information strategy

By correcting their digital footprint, in response to privacy tensions, consumers can manage immediate privacy threats. For example, they might *self-censor* or filter content after it has been published, by deleting content from blog entries or Facebook posts, “untagging” themselves in photos or posts, “unfriending” contacts, or requesting that a firm or social media platform remove their information. Consumers also might *avoid* disclosure by intentionally refusing to provide certain elements of information in response to initial requests (Martin & Murphy, 2017) or else *falsify* the information they do provide, such as using a fake name, address, date of birth, and profile picture. This strategy reduces their digital footprint by removing or altering content that previously has been available.

### Proactive information strategy

Rather than managing content that already has been published, a proactive information strategy uses *restraint* as a protective mechanism that defines consumers’ ongoing practices of withholding information (Lwin et al., 2007). Consumers reduce the amount of personal content shared, minimize digital interactions, and limit activities such as online check-ins, which can reveal personal information. They also might use *encrypted communications* such as Pretty Good Privacy software, S/MIME standards (Kaaniche et al., 2020), or anonymous re-mailers to reduce data availability. Some people seek *non-digital alternatives* for their communications, information search, and purchases (Martin & Palmatier, 2020). Since this strategy restricts information prior to sharing, it limits content and sociability. It also generally involves more effort, complexity, and inconvenience for consumers than a reactive information strategy, because it demands continuous monitoring of the digital footprint.

### Reactive permission strategy

In a reactive permission strategy, consumers limit access to their personal information when service providers ask for it or respond to an instant threat such as a data breach that makes the risk salient. Consumers generally might agree to provide access to their information, but with a reactive strategy, they engage in a *withdrawal* tactic to remove themselves from risky situations, such as deleting apps that ask for access to their location, rejecting or removing cookies from their computers, and blocking advertisements (Yap et al., 2012). A *fortification of identification* effort might include changing passwords after data breaches or threats. They also can minimize risk by *communication termination,* or opting out of firm communications to avoid intrusion and prevent third-party information access.

### Proactive permission strategy

Among consumers who are more aware of privacy tensions and knowledgeable about digital privacy technologies, we note more sophisticated efforts to protect personal information (Martin et al., 2017). With *screening*, they monitor their own digital activities by verifying firms’ privacy policies and securing transactions (e.g., using https protocols). *Restriction* involves limiting information access by adjusting privacy settings, such as turning off location-based access or changing cookie settings. *Identity masking* is another popular strategy to prevent tracking, using a security feature that stops a browser from storing cookies and the search history. Even more sophisticated tools include virtual private networks and The Onion Router, which work through encryption and create networks of virtual tunnels, designed to anonymize internet communications (Kaaniche et al., 2020). Finally, if they adopt *security consolidation*, consumers install privacy-enhancing technologies, such as blockers and firewalls for third-party trackers, along with internet security programs (Zarouali et al., 2020). These strategies offer strong protection but also require substantial technological savvy that is unlikely to be possessed by all consumers.

### Firm privacy responses

According to structuration theory, augmented by the SDL, firms as actors operate in broader systems that affect their behaviors (Vargo & Lusch, 2016). Firms are influenced by structure (e.g., regulations) and by their relationships with other actors (e.g., consumers) (Park et al., 2018). In response to regulatory and consumer actions, firms might comply with privacy rules (reactive response) or go beyond them to engage in privacy innovation (proactive response), which potentially shapes new structures (Luo, 2006).

#### Reactive response (privacy compliance)

Structuration theory (e.g., Luo, 2006; Park et al., 2018) suggests the presence of some structurally embedded constraints on actors. Due to increased scrutiny of data practices, firms are expected to comply with what is sometimes a patchwork of local, national, and international privacy regulations. A reactive response corresponds to the minimum expectation for a company, namely, to follow existing, immediate structures in the regulatory framework. With this *local approach to privacy* regulation, firms only aim to meet specific, local privacy rules. This type of response is common among small, local businesses, but it also might be adopted by big corporations, to take advantage of variances in legal systems across specific markets.

Furthermore, this approach is in line with a privacy process that emphasizes *privacy as a feature.* That is, privacy constitutes added value, generally included as an afterthought in product and service development efforts. The main goal underlying this approach is to stay within legal boundaries and general expectations related to privacy. For example, by strengthening their cybersecurity, companies can address consumers’ reactive information strategies by minimizing negative events such as data breaches that threaten to trigger consumers’ falsification, avoidance, withdrawal, or communication termination actions. In addition, these firms likely focus on technologies that enable them to adhere to regulations. When the GDPR came into force and required firms to ensure consumers’ right to be forgotten, they faced technological challenges and thus committed to developing automated and standardized procedures for the removal, transfer, or recovery of data, upon consumers’ request, which also might dissuade consumers from adopting self-censorship behaviors.

#### Proactive response (privacy innovation)

In a volatile business environment marked by constantly changing structural parameters, structuration theory suggests that firms can influence structural forces. For example, Xerox, Cisco, Nokia, and Motorola persistently and efficaciously convinced the Chinese government to update and require all firms to conform with a new set of industry technical standards, thereby changing industry norms as a key structural parameter (Luo, 2006). Privacy innovations are new or enhanced firm privacy management practices designed to benefit consumers, appease the government, or otherwise appeal to relevant stakeholders. They arise when firms actively integrate compliance as a business pillar and attempt to address privacy regulations collectively, through a *universal approach to privacy*. Instead of dealing with each law and policy separately, firms identify key compliance issues across regulatory frameworks and adopt a streamlined, uniform strategic plan that can guide all aspects of their behavior, as well as current and future standards.

Furthermore, privacy innovation encompasses a *privacy by design paradigm*, which embeds privacy in all business processes, products, and services, from their initial development to their final consumption and disposition stages (Bu et al., 2020). Privacy by design stresses proactive, user-centric, and user-friendly protection, and it requires substantial investments and changes. For example, data collection strategies would aim to gather zero-party data, which refer to consumers’ voluntary provision of their information, are completely consent-based, and can be collected from polls, quizzes, or website widgets (Martin & Palmatier, 2020). By engaging in data discovery, categorization, and flow mapping, innovative firms might minimize their information collection and only collect what they actually need. Privacy might be integrated into customer-facing applications too, such as automatic timed logouts, notifications for unrecognized access, and setting security and privacy as default options. Finally, privacy innovation encompasses accountable business practices that require firms to involve their partners in data governance to ensure end-to-end security, such as by auditing third parties that manage data on a firm’s behalf (Merrick & Ryan, 2019). Pursuing privacy innovation can address the proactive privacy responses of even highly skeptical consumers and instill trust, by creating a safe ecosystem, so it should minimize restraint and restriction behavior. In this sense, privacy innovation offers an effective way to address both proactive consumer responses and regulations. However, it also tends to be costly and requires both long-term commitments and extensive transformations of the business structure and practices.

In summary, structuration theory purports that a privacy-related structure must include regulations that require firms to provide notice and gain consent from consumers to collect, parse, and store their data. They greatly enhance consumer-initiated strategies to address technology–privacy tensions. Consumers’ behaviors also depend on their resources, such as knowledge and self-efficacy (Walker, 2016). In general, reactive strategies require less expertise, and proactive ones demand greater technological savvy. Yet firms remain bound by the structure and can employ either a reactive response that treats privacy as a compliance issue or a proactive response that views it as a core business value. These trade-offs and tensions characterize regulatory–consumer–firm interactions, and we rely on them to propose an integrated framework to inform theory, practice, and policy.

## Integrated framework of the structuration of privacy

**Fig. 1:**
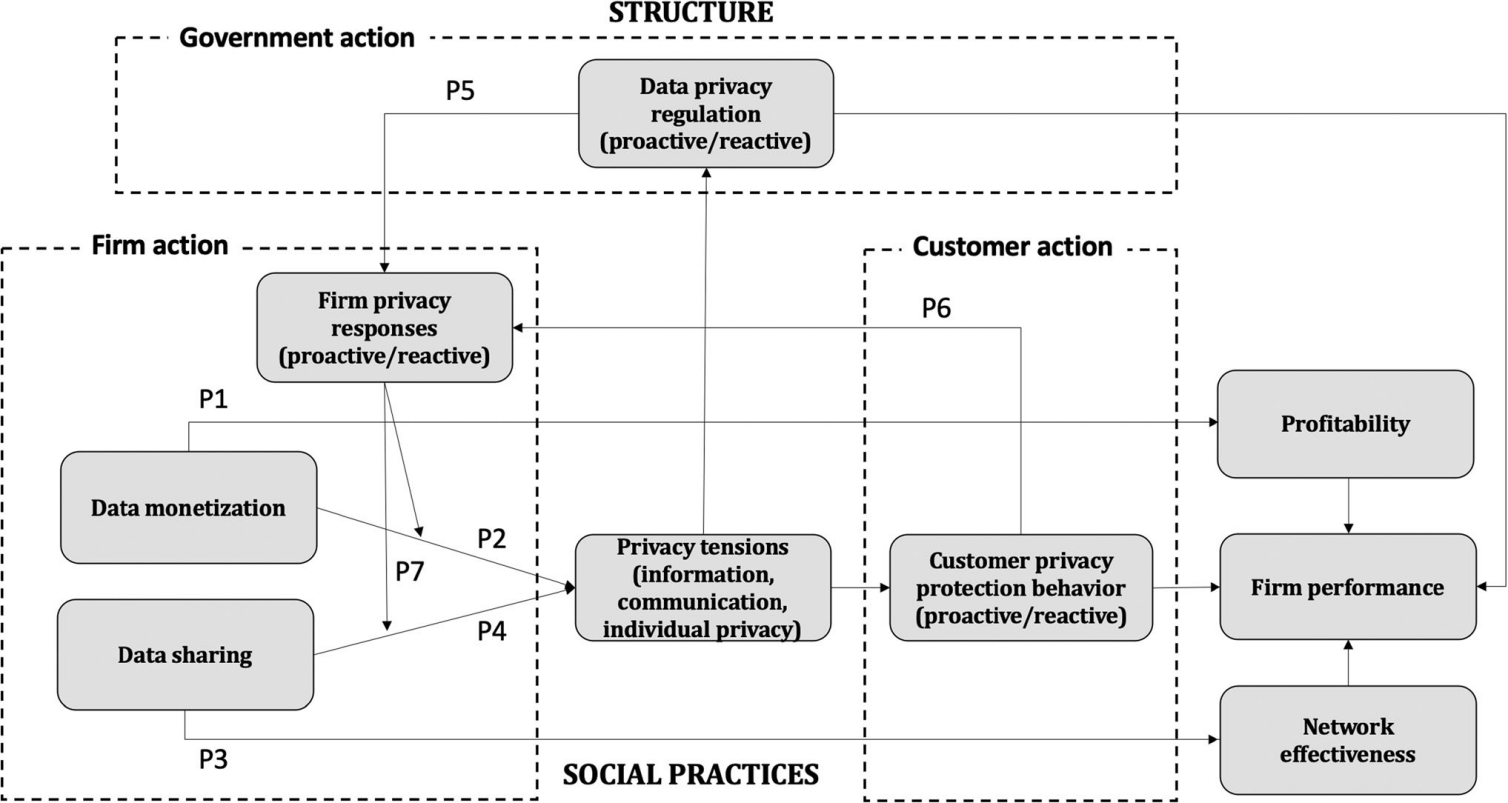
Integrated framework of privacy structuration

The preceding review offers key insights and implications for firms, consumers, and regulators. Informed by structuration theory, and augmented by elements of SDL, we draw from these insights to develop an integrated framework (Fig. 1), in which privacy and its preservation emerges from interactions across structures (i.e., digital technologies as resources and data privacy regulations as rules) and actors (i.e., firms’ and consumers’ actions). On this basis, we propose a series of tenets related to themes of (1) data monetization and firm performance, (2) data sharing and firm performance, and (3) firms’ data privacy actions. We also introduce associated propositions. This synthesis of extant literature reveals practical insights to clarify the future of digital technologies in contexts marked by changing consumer behaviors and regulatory parameters. Depth interviews and case studies (Table 3) provide additional, conceptual scaffolding to proposed tenets and propositions, in support of our framework.

**Table 3:** Integrated data strategy framework and case studies

Company and Sources	Tenets and themes	Data Strategy	Effect on Firm Performance
***Facebook*** Patterson (2020); FTC (2019); Lapowsky (2019); Shapiro (2019); Weisbaum (2018); Wong (2018)	***Tenets 1 and 2*** Data monetization; Data sharing; Privacy regulation; Privacy risks; Customer privacy protection behavior; Firm performance	Facebook extensively monetizes user data through extended data wrapping, such that it provides data analytics-based features to its clients (e.g., advertisers), for example, targeted advertising based on users’ activity, and measuring the ad effectiveness by tracking users’ digital footprints. Facebook shares substantial data with partners such as app developers; it had allowed third-party apps to access data on Facebook users’ friends for years, which led to an infamous scandal in which Cambridge Analytica acquired data on millions of customers to build comprehensive personality profiles without their knowledge in 2018.	Data monetization fuels Facebook’s profitability. In 2018, the value of Facebook users’ personal information was equal to $35.2 billion, or 63% of Facebook’s revenues. However, Facebook has come under scrutiny due to its data practices. After the Cambridge Analytica scandal, Facebook was fined US$5 billion by the Federal Trade Commission, and £500,000 by the UK’s Information Commissioner’s Office for their role in the scandal. The event sparked heated debates about consumers’ privacy rights, prompting policy makers to increase the stringency of data regulations. The privacy scandal resulted in a decrease in overall trust in the company, falling daily active user counts in Europe, and stagnating growth in the US and Canada.
***Apple*** Apple (2021); Leswing (2021); O’Flaherty (2021)	***Tenets 1 and 3*** Data monetization; Proactive privacy responses; Privacy risks; Firm performance	Apple uses digital technologies to gather and make sense of data for internal monetization purposes, such as optimizing marketing and business performance, developing prediction analytics to improve user experiences, and innovating new products and services. Apple also engages in data wrapping, such as through the Apple Health App, which tracks users’ physical activities and biometrics and create alerts if health issues arise. Apple shares data with partners such as suppliers and app developers. Apple has adopted privacy-by-design principles and used enormous digital resources to develop privacy innovations, such as Intelligent Tracking Prevention in Safari, Privacy Labels on the App Store, and App Tracking Transparency.	Apple performs exceptionally; its revenues soared by 54% to $89.6 billion in the first quarter of 2021. While engaging in monetization practices, privacy initiatives have reduced the perceived risks of using Apple products and positively influenced customer responses. More than two-thirds of Apple customers agree with its privacy policies and 92.6% of Apple users stating they would never switch to an Android. The App Tracking Transparency privacy innovation encourages advertisers to use Apple’s own Search Ad in the App Store, further strengthening the impact of data monetization on firm performance. This data privacy innovation thus is changing industry norms, shaping new customer privacy behaviors, and reinforcing existing data regulations.
***BMW*** BMW (2021); Nica (2020); Wilkie, 2020	***Tenets 2 and 3*** Data sharing; network effectiveness; firm performance; data privacy regulation; proactive privacy responses	BMW has engaged extensively in data sharing and but imposed strict limits on how those data can be used for monetization. To detect and rectify product defects, it is essential for its partners and suppliers to obtain data assigned to a specific vehicle, on a case-by-case basis. BMW has adopted innovative privacy approaches, including pseudonymization to encode personal information, that establishes smooth procedures while preventing other parties from tracking customers. In 2020 BMW and automotive manufacturers and suppliers, dealer associations and equipment suppliers joined a data-sharing alliance to build a cloud-based data exchange platform.	Data sharing enhances the effectiveness of the business network, which improves BMW’s performance. It can proactively monitor product functions, increase value chain efficiency, and enhance customer experiences. Data sharing enables BMW and its suppliers, to pinpoint production bottlenecks or parts shortages, which can boost in-network effectiveness and the performance of all firms involved. The new cloud technology is designed with privacy and security in mind, allowing European car manufacturers to maintain control over their own data. This initiative helps them formulate effective responses to potential scenarios, such as the coronavirus lockdown that imposed serious pressures on the supply chain.

To verify our propositions relative to firms’ and consumers’ experiences with digital technologies and data exchanges, we conducted in-depth interviews with ten senior managers in various industries, with 4 to 31 years of experience in their respective areas. We also interviewed five consumer informants from 27 to 41 years of age who are heavy users of digital technologies (see Web Appendix 2 for informant profiles). We identified participants from our contacts in a research cluster. Interviews were conducted either face-to-face or via a video conference platform, and they were recorded and transcribed. The interview protocol includes 18 questions related to digital technologies, data collection and use, and privacy issues (see Web Appendix 3).

### Tenet 1: Data monetization and firm performance

We propose that digital technologies function as resources that enable firms’ data monetization and data sharing strategies. Using digital technologies such as big data, IoT, and AI in the ways previously described, firms can convert data and analytics into value for their customers and increase their profitability (Najjar & Kettinger, 2013). For example, a recent estimate of the value of Facebook users’ personal information is $35.2 billion, or 63% of Facebook’s revenues (Shapiro, 2019). As a shared general consensus, the interviewed senior managers agreed that data analytics boost firms’ performance. Internal data monetization practices can enhance firm performance, because the data collected from digital platforms represent consumer insights that firms can use to tailor solutions to meet consumers’ preferences and also make better business decisions (Bleier et al., 2020). As the head of product marketing in an electronics firm noted: “By using data we can offer the right product, right value at the right touchpoint to the end-user [using the] right approach.” An informant who performs customer analytics in the banking and finance sector also provided an example of data wrapping practices, such that the organization packaged its products with data insights as value-added features:

[Some of the data] that we capture [from individual customers] can be used to provide insights to our B2B customers…. With what we have today we could provide insights into their business based on their data to help them grow their business.

Similarly, external monetization, such as selling data to clients for marketing and targeting purposes, offers significant economic benefits for sellers. These three approaches are not mutually exclusive; firms can use more than one to generate revenue. Such data monetization practices increase the profitability of a firm and thereby enhance its performance.

Privacy tensions can stem from consumer–firm interactions through digital technologies (Park et al., 2018). Drawing from the notion in structuration theory that structure can both shape and be shaped by social practices, we note that the inherent privacy tensions of data monetizing practices provoke consumer and regulatory privacy responses, which have direct implications for firm performance. Data monetization thus may lead to privacy tensions and open firms to legal challenges, especially as data privacy regulations grow stronger. After the Cambridge Analytica scandal, heated debates about consumers’ privacy rights arose, and policymakers sought to increase the stringency of data regulations, such that Facebook’s CEO was called to testify before Congress and the company was fined US$5 billion by the U.S. Federal Trade Commission for deceiving users about their ability to control the privacy of their personal information (Lapowsky, 2019). In addition, trust in Facebook plunged by 66% (Weisbaum, 2018) and customers, including influential figures such as Elon Musk, joined the #DeleteFacebook movement in response. As this example shows, monetizing data may spark consumers’ privacy protection behaviors, which can jeopardize firms’ relationships with them. Even requests for data or perceptions that firms profit from consumer data can trigger reactive and proactive privacy protection behaviors, such as information falsification or outright refusal (Table 2). One consumer informant recalled an experience that felt like “an invasion, like I visited a website once because we got a new kitchen and now I get ads constantly for kitchen stuff. And it’s like, I might need that, but I don’t want you to know that I need it, but I want to find it myself.” A senior manager, head of digital marketing for an apparel firm, echoed this sentiment by acknowledging that “society is a lot more worried about data.” The inherent privacy tensions of data monetization can increase regulatory scrutiny, damage customer–firm relationships, and spark consumer privacy protection behaviors. We propose the following tenet and propositions:

#### Tenet 1 (Data Monetization Trade-Off)

Enabled by digital technologies, data monetization creates a trade-off between firm profitability and privacy tensions (information, communication, and individual privacy). When they result from consumer–firm interactions, privacy tensions lead to changes in both regulatory and customer responses.

#### Proposition 1

Data monetization positively influences firm performance through profitability.

#### Proposition 2

Data monetization negatively influences firm performance through increased privacy tensions (information, communication, and individual privacy), which trigger consumer data protection behaviors and privacy regulations.

### Tenet 2: Data sharing and firm performance

From the integration of structuration theory and the SDL, we determine that digital technologies enable data sharing among actors within a business network, so multiple parties can access the data, anytime and from anywhere, which increases efficiency, in line with the prediction that value is co-created by multiple actors in an ecosystem (Vargo & Lusch, 2016). Data sharing also strengthens relationships among supply chain partners and fuels network effectiveness, which refers to the “viability and acceptability of inter-organizational practices and outcomes” (Sydow & Windeler, 1998, p. 273). Firms might collectively improve their performance by complementing their data with others’ information, thus generating second-party data (Schneider et al., 2017). Manufacturers gather market analytics from distributors for new product design, demand forecasts, and the development of marketing strategies. Take the automobile industry as an example. Data sharing enables carmakers, including BMW and its suppliers, to pinpoint production bottlenecks or parts shortages, then formulate effective responses to potential supply chain problems and boost the performance of all firms involved (BMW, 2021). A chief financial officer of a manufacturing firm affirms the value of data sharing:

Definitely, you know, for us as a supplier when we receive our clients’ market data, that’s entirely valuable for us. [Data sharing] is a critical part of making sure that we do the best job that we can.

Yet similar to data monetization, the multiple-actor, collaborative nature of data sharing can result in privacy tensions. The more data a firm shares, the more control it must surrender to other parties, creating vast uncertainty. Therefore, data sharing may jeopardize consumer information privacy and trigger both consumer and regulatory responses. These responses may imply performance losses for the focal firm, especially if requisite security measures are missing (Schneider et al., 2017). Considering the interactions between structure and social practices of data sharing, we offer the following tenet and propositions:

#### Tenet 2 (Data Sharing Trade-Off)

Enabled by digital technologies, data sharing creates a trade-off between network effectiveness and privacy tensions (information, communication, and individual privacy).

#### Proposition 3

Data sharing positively influences firm performance through network effectiveness.

#### Proposition 4

Data sharing negatively influences firm performance through increased privacy tensions (information, communication, and individual privacy), which trigger consumer data protection behaviors and data privacy regulations.

### Tenet 3: Firm privacy responses

The dynamic interplay of structures and actors, again, guides our theorizing. Privacy tensions that occur from data monetization and data sharing (i.e., social practices) tend to alert policymakers, who often respond by strengthening regulatory frameworks. This change in structure (i.e., data regulation), together with objections from consumers that result in privacy-protective behaviors, requires firms to develop data privacy actions to reduce privacy tensions. Firms that aspire to address privacy tensions proactively must devote resources and create processes for updating their digital technologies, which is a particular challenge for small firms: “I think we would like to focus more on innovation. But we’re probably not that big a company at the moment that we can put a lot of resources towards it” (head of digital marketing, apparel).

A common centerpiece of privacy legislative frameworks is an emphasis on consumer consent. Adhering to such practices may reduce the collection and use of data and limit the number of parties with which data can be shared, which can engender diminished firm performance due to the constraints on personalization, customization, targeting, and prediction efforts. In addition, privacy laws might restrict firms’ ability to sell data and analytics and increase legal expenses, causing a significant disadvantage for businesses that are unable to collect data on their own. However, the effects of data privacy regulation can be mitigated by proactive privacy responses. Technology innovations might be exploited to circumvent regulatory limitations. For example, Airbnb and Uber personalize predictive models without compromising consumer privacy by using smart pricing algorithms based on anonymized, aggregated, or market-oriented (event- or object-based) data, rather than personally identifiable information (Greene et al., 2019). Whether they enhance privacy practices or technological processes, privacy innovations allow firms to reap critical data benefits, meet or exceed legal and regulatory obligations, accommodate consumer expectations, reduce privacy tensions, and, ultimately, mitigate the impacts of consumer responses and regulation stringency. Even though they involve data monetizing for marketing, operational efficiency, and data wrapping practices, Apple’s privacy initiatives (e.g., App Tracking Transparency, Privacy Nutrition Labels) have boosted customers’ loyalty to the brand even higher, such that 92.6% of Apple users claim they would never switch to an Android (O'Flaherty, 2021). The initiatives also alter industry norms and impose greater pressure on competitive firms, such that Google has announced plans to consider an anti-tracking feature for Android devices (Statt, 2021). As a chief executive officer of a telecommunication service provider explained:

You certainly got to comply, ticking that box, but I do think that if you were going that extra mile and looking at ways [of] being innovative, then you’re going to be servicing your customers even better.

We offer the following tenet and propositions:

#### Tenet 3 (Firm Privacy Responses)

Enabled by digital technologies, firms develop privacy responses to address regulatory and consumer privacy responses.

#### Proposition 5

Data privacy regulation can both positively and negatively influence firm privacy responses.

#### Proposition 6

Consumer privacy protection behaviors positively influence firm privacy responses.

#### Proposition 7

Compared with reactive privacy responses, firm proactive privacy responses reduce consumer privacy protection behavior by mitigating the negative effects of data monetization and data sharing strategies on privacy tensions.

## Implications for firms with different data strategies

Our novel framework explains that digital technologies empower firms’ data monetization or data sharing strategies, while also creating privacy tensions. Although most firms seek vast customer data and employ various means to leverage them, not every firm requires the same amount of data or has the necessary digital technology capabilities (see Web Appendix 1). The effect of data privacy regulation on data sharing and monetization thus should vary across firms which rely on data to varying levels. The more firms rely on data monetization and data sharing, the more pronounced the effects of regulatory changes and consumer data protection behaviors become. For such companies, data privacy responses have heightened implications; a proactive response might mitigate the restrictions. We apply our framework to provide implications to firms with various data strategies as depicted in Web Appendix 1, in which we assign firms to one of four groups, according to their levels of both data monetization and data sharing practices.

### Data harvester

Data harvesters engage in limited, internal data monetization and data sharing practices, leaving them less exposed to consumer privacy behaviors and regulations. Many of them seek to “harvest” their own data to create customer value. The head of digital marketing of an apparel firm shared:

We don’t have as much access to that sort of [third-party] data but yet we do have first-party data, which will be those [data] of those people within our leads database in our customer base.

However, if restrictions were imposed on their internal consumer data collection and use, they would have to rely on third-party data providers or brokers. Therefore, data harvesters likely comply closely with government regulations and tend to adopt reactive privacy strategies to protect consumer privacy.

### Data patron

Data patrons’ monetization approaches resemble those of data harvesters, such as developing customer intelligence for better internal marketing efforts. However, data patrons such as Apple, Microsoft, and PayPal are more likely to undertake data wrapping to create customer value, because they have greater digital technology capabilities. In addition, they are cautious about the many partners involved in their business networks which can create significant risks for sharing practices. Echoing this view, the head of customer analytics of a banking and finance firm stated:

We do have some partnerships that we share [our data with], but again it depends on our terms and conditions on what we share and what it is related to, especially if it has to do with the customer experience and if it is in the right interest of our customer.

These firms are moderately affected by consumer privacy behaviors and regulatory frameworks. Innovative patrons can navigate sophisticated regulatory frameworks and consumer privacy protection behaviors; for example, Apple’s App Tracking Transparency promotes its image as a responsible tech firm.

### Data informant

Data is the bloodline of data informants’ business models, as described by the president of a software development firm: “We don’t produce any physical goods. We operate only in the informational space. That means data is everything.” To maximize profits, some data informants use questionable methods to identify people’s interests in sensitive topics. These firms face substantial scrutiny; some regulators suggest they should be listed in public registries and allow consumers to request clarifications about data ownership. Their size and scope also make these firms prime targets for cyberattacks (Bleier et al., 2020). Finally, their privacy innovation tends to be low, because few incentives (or punishments) limit their data exploitation.

#### Data expert

Data experts are active members of the digital technology ecosystem and work with any third parties, engaging in very high levels of both data sharing and data monetization. Other firms rely on data experts for advertising insights and customer analytics, so in turn, they have significant power and influence over the nature and amount of data collected. Only a few firms (e.g., Google, Facebook, Twitter) fit this description, and each of them is subject to ongoing regulatory scrutiny. In general, stronger regulations, competitive maneuvers (i.e., Apple’s iOS 14 privacy updates affecting Facebook, Twitter, and others), and increasing consumer criticism may threaten their business model. Accordingly, data experts may need to adopt more extensive, transparent, and proactive privacy practices to address these challenges.

## Recommendations for policymakers

Privacy regulations attempt to empower consumers by ensuring their ability to share, capably monitor, and protect their personal data. Regulations also provide guardrails to constrain firms’ interactions with consumers by mandating responsible data use and fair exchange. On the basis of our integrative framework and typology of data strategy, which establish a comprehensive view of privacy tensions linked to emerging digital technologies, we offer policymakers several recommendations for drafting, implementing, and monitoring effectively such regulations.

### Addressing digital technology evolution

Digital technologies evolve rapidly, and privacy regulations must account for that rapid evolution. Although future-proofing privacy regulations is untenable, regulatory parameters that govern fundamental data exchanges, rather than specific technological techniques for gathering or processing data, can protect consumers more broadly, even as technologies change. In addition, such regulations would prevent firms from applying technologically advanced workarounds to subvert the restrictions. Both the GDPR and CPRA are designed to be technology neutral, governing the data exchanged between a customer and a firm rather than the technology through which the data are exchanged. Nevertheless, emerging digital applications such as deep fakes, the rampant spread of misinformation, and the growth of advertising ecosystems can challenge even the most technologically broad regulatory mechanisms. It is thus imperative that regulatory frameworks adequately protect consumer data, regardless of technological advances, with legal and protective parameters drafted with a technology-neutral approach that avoids regulatory obsolescence and prevents innovative subversion by firms.

Beyond imposing constraints though, we also recommend that regulators work closely with firms to learn how the regulations they propose are likely to play out in practice. For example, novel technologies can make the enforcement of various privacy regulatory dimensions more or less effective; requiring customer consent is a cornerstone of the GDPR framework, but its operationalization and enactment in practice has led to increased consumer annoyance with the required pop-ups (Fazzini, 2019). Monitoring efforts also should go beyond identifying violations or demanding strict, high-level compliance. In summary, even if technology advances too quickly to be subject to specific regulation, it strongly influences the implementation and effectiveness of regulation in practice, such that it can support or hinder intended regulatory purposes, so policymakers need to pursue and maintain an up-to-date, clear understanding of recent technology developments.

### Appreciating variability across firms

The GDPR may have had the unintended consequence of empowering the big technology companies (Facebook, Google) that it originally sought to constrain (Lomas, 2020). Large companies with many resources (financial, legal, personnel) are better poised to accommodate vast regulatory changes, including privacy regulatory mandates. In particular, firms that already house vast troves of customer data easily can reduce their reliance on external or third-party data providers. Their in-house data capabilities enable them to conform with regulatory parameters, even if their data use might seem ethically questionable. We recommend that regulators examine firms’ specific data sharing and data monetization practices closely, with particular monitoring efforts focused on firms with extensive engagement in data monetization, such as data informants and data experts. This targeted means to privacy regulation avoids some of the weaknesses of a “one-size-fits-all” approach. For example, many data informants are developers that offer free apps in exchange for customer data, and their external monetization practices are largely unknown to consumers. By applying the proposed data strategy typology, policymakers can detect areas of data concentration with the potential for misuse, such as among data experts and data patrons. Such considerations also could help limit the dominance of major tech firms. Regulators might apply the typology to understand the adverse effects of privacy regulation, such as data portability on data harvesters, that might harm small businesses or start-ups. If they cannot acquire large troves of data on their own, these smaller competitors must rely on larger actors to obtain data-based insights.

### Promoting proactive regulatory enforcement

To the extent possible, regulatory frameworks should impose proactive enforcement of both privacy policy requirements and managerial practices, including privacy by design and privacy by default principles. Monitoring a firm’s data protection behavior can indicate the effectiveness of the regulatory framework, beyond just capturing violation occurrences or noncompliance. Even with comprehensive data protection regulations in force, multinational corporations appear to adopt reactive privacy strategies for the most part, while continuing to engage in behaviors and practices that put them and their customers at risk for data breaches (Norwegian Consumer Council, 2020). By recognizing and rewarding proactive firm responses (data innovation), perhaps in collaboration with industry bodies or aspirational firms, regulators also could encourage the reproduction of best practices.

Finally, transnational cooperation among enforcement authorities is necessary to deal with the many multinational firms and online businesses whose operations transcend national borders. Harmonization efforts proposed by the United Nations provide a potentially useful platform; its Personal Data Protection and Privacy Principles can help member organizations navigate the diverse patchwork of regulatory coverage, given their business scope and reach, by developing a coherent set of widely applicable rules that embody strong, proactive standards. Effective enforcement of privacy regulation and true protection of consumer privacy can be realized only if globally cooperative mechanisms are in place.

## Conclusion and research directions

Adapting to ever-changing business environments and developing long-term relationships with key stakeholders requires extensive investments in digital technologies. Enabled by digital technologies, modern firms have access to massive amounts of data, which they use to pursue various advantages. This research provides new insights into the role of digital technologies by adopting a multidimensional approach and synthesizing current research and practical insights from the perspectives of firms, consumers, and regulators—each of which is critical to developing an integrated, comprehensive framework.

We begin by identifying the pressing tensions between firms’ data monetization and sharing practices enabled by digital technologies, as well as their implications for consumer privacy. By leveraging structuration theory, infused with elements of the SDL, we delineate responses to these tensions exhibited by regulators, consumers, and firms. The intersection of the firm, consumer, and regulatory perspectives produces an integrated framework of three tenets and seven corresponding propositions with the potential to advance understanding of privacy as a central product of the interactions across structure (i.e., digital technologies and regulatory frameworks) and social practices (i.e., firms’ and consumers’ actions). We overlay these predictions with a typology of firm strategies for data monetization and data sharing to highlight how firms’ privacy responses are influenced by resources (i.e., digital technology), rules (i.e., regulations), and other actors’ actions (i.e., consumers’ privacy protection behaviors).

To stimulate further research, we propose an agenda across three broad areas: firm data and privacy strategies, regulatory impacts on firms and ecosystems, and consumer responses to the digital technologies. Each area comprises multiple research questions and avenues for marketing researchers and practitioners, which we summarize in Table 4.

**Table 4:** Research agenda for data strategies

Theme	Brief Description	Research Questions
**Firm data and privacy strategy**		
*Data strategy*	As data become the new currency in the digital era, the firms that can create unique and sought-after data and business intelligence from data-generating technologies wield increasing power. Firms need to maximize value from data by creating balanced, responsible data monetization and data sharing.	• What data valuation models can motivate responsible data monetization and risk minimization? • How do changing work cultures, such as increasing uses of home networks, personal and shared computers, and access to a wide range of systems from outside the office, increase the threat of cyberattacks or data breaches? • Can data sharing be improved via data interoperability?
*Privacy innovation*	The benefits of data privacy innovations require further investigation. In particular, additional research is needed to identify effective data privacy innovations that might enhance the outcomes of data sharing or data monetization, as well as the challenges to the adoption and implementation of privacy innovations.	• Will firms’ enhanced data privacy practices become the new standard, such that innovating firms need to be even more novel in their privacy practices? • How is the relationship between innovative firms and digital technology providers likely to evolve? • What is the sustainable level of investment in privacy innovation for data harvesters, data informants, data patrons, and data experts? • Do the benefits of privacy innovation justify the costs of adoption and implementation?
**Regulatory impact on firm level and ecosystem level**		
*Impact of privacy regulation at firm level*	According to structuration theory, structure is both the context and product of actor behavior. In other words, firm responses to privacy regulations can shape the regulatory framework, which in turn constrain or promote subsequent firm actions. The impact of regulations may vary among firms with different characteristics such as data strategy and firmographic variables.	• What are the risks of firms failing to adhere to reactive and proactive privacy requirements? How do differences in regulations moderate these effects? • To what extent do firm responses to regulatory frameworks change the regulation itself, which in turn might trigger a different set of firm actions? • How does the data strategy type moderate the effect of privacy regulation on firm performance? • How do firmographic variables moderate the effect of privacy regulation (stringency) on firm performance?
*Impact of privacy regulation at network or ecosystem level*	Privacy regulations may be increasingly necessary, but also threaten the benefits that firms and consumers receive from data monetizing and sharing. That is, greater limits clearly may be warranted, but more stringent regulations can create unintended obstacles to value co-creation in a firm’s network or broader ecosystem, especially when there may be fragmentation in legislative regimes.	• What is the impact of regulation stringency on network performance? Does this effect vary with industry settings? • How do policy makers balance consumer privacy protection with industry innovation? • Do the effects of privacy regulation on firms that control flows of data, such as data experts and data patrons, spill over onto firms that are reliant on external data, such as data harvesters? • How do interactions of sector-specific regimes influence ecosystem performance?
**Consumer responses to the new digital era**		
*Privacy attitudes*	As digital technologies penetrate consumers’ lives and new technologies become powerful means for firms’ data collection and use, it is important to understand consumers’ attitudes toward privacy in response to firm and regulatory actions.	• What are consumers’ attitudes toward firms implementing privacy innovations and firms using a specific data strategy (i.e., data harvesters, informants, patrons, and experts)? • Do new technologies, such as super AI, threaten consumer information, individual, and communication privacy? • How do consumers respond to regulation stringency? • What can be done to make data trade-offs more acceptable for customers?
*Privacy protection behavior*	In response to emerging threats to privacy, consumers employ various forms of protection, both reactive and proactive. Understanding when these responses are triggered can help firms devise effective strategies.	• What are the outcomes of consumer privacy responses, and how do they translate into financial impacts on firms? • When do consumers activate reactive and proactive responses? • What role do privacy-enhancing technologies play in consumers’ privacy responses?

### Firm data and privacy strategies

#### Data strategy

Our proposed framework acknowledges the reality of growing data monetization and data sharing practices, as focal strategies that determine firm performance. Data are the new currency in the digital era, and firms that can create unique, sought-after data, business intelligence, and responsible data-generating technologies will wield increasing power. To maximize the value of data, firms should seek balanced, responsible data monetization and data sharing. Such efforts would benefit from an optimal data valuation model that promises to maximize value and minimize risks through responsible data monetization and sharing. Firms should carefully consider their dynamic inventory of information assets, the features of their data that are central to realizing their potential, and metrics for assessing the value of data and returns on their investments (Deloitte, 2020).

Data sharing poses unique challenges, especially across platforms and organizations. A key question is how to improve data sharing through interoperability on secure, permission-based platforms. In addition, changing work cultures, such as the increased use of home networks, personal and shared computers, and access to office systems from external work venues, increase threats of cyberattacks and data breaches. Such developments represent new obstacles to data sharing, and they offer a fruitful research area.

#### Privacy innovation

Regardless of firm size or data strategy type, data privacy innovations extend beyond mere compliance and can lead to competitive advantages. The benefits of data privacy innovations across all firm data strategy types require further investigation. In particular, data privacy innovation should gain momentum as a positive catalyst for the firm and ecosystem performance. We need research that details effective implementations of privacy innovation practices and their long-term effects.

Research also is needed to identify effective data privacy innovations that might enhance the outcomes of data sharing and data monetization. In particular, research might determine sustainable levels of investment in privacy innovation, required for firms of different sizes, business models, and data strategies (i.e., data harvesters, data informants, data patrons, and data experts). A key question is whether the benefits of privacy innovation justify the cost of its adoption and implementation. For example, state-of-the-art security schemes and privacy-preserving technologies (e.g., blockchain-based approaches) still suffer disadvantages regarding scalability and data storage limitations (Jin et al., 2019), and they remain costly to implement, despite the data protection improvements they provide.

Moreover, privacy innovation might create ripple effects, due to the interconnected nature of firms in an ecosystem. For example, privacy changes among data experts and informants might influence data harvesters, who rely on the services of those data experts and informants. Therefore, a potential research consideration might be the ripple effects of privacy innovation, including the positive inspiration of an ecosystem revolution but the simultaneous strain they put on the relationship between firms and other actors.

### Regulatory effects on firms and ecosystems

#### Effect of privacy regulation on firms

We anticipate that as regulatory frameworks and consumer responses to privacy issues evolve, firms will face more restrictions on their strategies and practices. Continued research should examine the risks if firms fail to adhere to reactive and proactive privacy requirements. The outcomes might be subject to contextual factors, because regulation stringency varies across countries and industries. Privacy regulations restrict firm actions related to data monetization and sharing, but as we have outlined, structuration theory also predicts that actors’ behaviors shape the structures. Therefore, it would be interesting to identify the extent to which firm responses to regulatory frameworks can change the regulations themselves, which in turn might trigger a different set of firm actions. Uncovering nuanced effects according to firm size, industry, and other firmographics is an important direction to inform regulatory efforts. We thus call for investigations of how the typology of data strategy and firmographic variables moderates the effect of privacy regulations on firm performance.

#### Effect of privacy regulations on firm networks

Privacy regulations may be increasingly necessary, but they also have the potential to restrict the benefits that both firms and consumers receive from data monetizing and data sharing. That is, greater limits may be warranted, but more stringent regulations can create unintended obstacles to the performance of a firm’s network and ecosystem. Research should continue to address the impact of regulation stringency on network effectiveness. Finally, different, fragmented data access regimes exist in various sectors, such as utilities, automotive, finance, and digital content/services (Graef & van den Boom, 2020). It is therefore relevant to test the interactions of sector-specific regimes to predict broader network performance.

### Consumer responses to digital technologies

#### Privacy attitudes

As digital technologies penetrate consumers’ lives further and become increasingly powerful means for firms’ data collection and use, fresh privacy concerns emerge. Research is needed to understand how new digital technologies such as artificial general intelligence (i.e., AI on a par with human intelligence) threaten consumers’ information, individual, and communication privacy risks. Research thus might investigate consumers’ perceptions of firms’ data strategy (i.e., data harvester, informant, patron, and expert), actual data privacy actions, and regulation stringency, which could help construct long-term, technology-proof outcomes for all parties involved. Notably, all our informants acknowledge data skeptics, who proactively protect their privacy by using encrypted platforms or refusing all personalized services, but they believe most consumers are willing to accept some loss of privacy in exchange for personalization of the right experience. A critical question is how to identify consumer segments and make data trade-offs more acceptable for them.

#### Privacy protection behavior

Research on privacy in marketing has predominantly focused on privacy concerns, leaving a dearth of evidence about actual privacy behaviors (Pomfret et al., 2020). Consumers employ various forms of protection from privacy risks, whether reactive or proactive. Understanding when these responses are triggered and the role of privacy-enhancing technologies in evoking them would help firms devise more effective strategies. Additional research could explore how consumer privacy responses translate into financial impacts for firms.

As our explication of digital technologies highlights, data and their applications continue to transform the marketing landscape. The novel framework we propose can help clarify digital technologies and the future of data for firms, consumers, and regulators. But much work remains. We hope this research offers some compelling directions for further inquiry, along with new insights into the future of digital technologies.

## Supplementary Information

ESM 1 (DOCX 104 kb)
