# Supplementary material for: Digital technologies: tensions in privacy and data
Source: J Acad Mark Sci. 2022 Mar 5;50(6):1299–323. doi: 10.1007/s11747-022-00845-y (PMC8897618; doi:10.1007/s11747-022-00845-y)
Supplement: Supplementary file 1 — (DOCX 104 kb) [file 11747_2022_845_MOESM1_ESM.docx]

**Web Appendix for**

**“Digital Technologies: Tensions in Privacy and Data”**

Web Appendix 1. Firm Data Strategy Typology

Web Appendix 2: Interview Script

Web Appendix 3: Depth Interview Informant Profiles

**Web Appendix 1. Firm Data Strategy Typology**

**Web Appendix 2: Interview Script**

**Firms**

***Digital Technologies***

1. What kind of digital technologies does your company use? How do you categorize digital technologies?
2. What is the role of data in your company? How does your company gather customer data?

***Sharing Data***

1. Does your company share the data with any party within your network partners (e.g., clients, distributors, business partners, etc.)? What are the sharing practices?
2. Do you think it is important to share data? Who would benefit from data sharing?
3. What type of companies require a lot of data sharing?
4. Do you think it is important for your business partners to share their data with you?
5. Is there any risk associated to sharing data? Any privacy issues?

***Profiting From Data***

1. How does your company use customer data?
2. Do you think that customer data usage can improve the profitability of your company? If so, in which way?
3. Do you notice any way a company can make money from customer data?
4. Is there any risk associated to using customer data? Any privacy issues?

***Privacy Responses***

1. What could be customer responses to privacy risks? How do they affect your business?
2. What are the regulatory responses in relation to privacy issues? How do they affect your business and industry?
3. How does your company respond to privacy regulations such as GDPR?
4. What is the privacy policy of your company? How was your privacy policy developed and implemented? Does it follow any particular privacy regulatory framework?
5. Any technologies can be used to improve privacy protection?
6. Do you think which one is more important - privacy compliance or privacy innovation?
7. What is your view in relation to customer data security and privacy?

**Consumers**

***Digital Technologie***s

1. What type of digital technologies have you used?
2. What kind of data do you provide when using these technologies?
3. What type of data do you provide to businesses?

***Sharing Data***

1. Do you notice any businesses sharing your data with others such as their partners? Which one do you think are more likely to share data?
2. Do you think it is necessary for companies to share your data? Are there any benefits?
3. Are there any risks associated with businesses sharing your data? What are they?

***Profiting from Data***

1. In which ways your data might be used by businesses?
2. Do you think that your data can improve the profitability of a company? If so, in which ways?
3. What types of businesses most benefits from your data?
4. Is there any risk associated to your data being used by businesses? What are they?

***Privacy Responses***

1. What are some of the potential privacy issues that you are concerned with?
2. How do you responses to these risks?
3. What are the regulatory frameworks that you notice? (e.g. GDPR)
4. Do you think the regulations/laws from the government are sufficient? Why?
5. Do you think firm actions regarding privacy protection are sufficient? Why?
6. Do you think which one is more important - privacy compliance or privacy innovation? Why?
7. Top of your head, do you recall any company that is doing extremely well with privacy protection? How about the worst? Why?
8. What is your view in relation to data security and privacy?

**Web Appendix 3: Depth Interview Informant Profiles**
